# Comparison of patients admitted to an inner-city intensive care unit across 3 COVID-19 waves

**DOI:** 10.1097/MD.0000000000033069

**Published:** 2023-02-22

**Authors:** Sindhaghatta Venkatram, Arundhati Dileep, Ked Fortuzi, Nishant Allena, Gilda Diaz-Fuentes

**Affiliations:** a Associate Professor of Clinical Medicine, Division of Pulmonary and Critical Care Medicine, BronxCare Health System, Bronx, NY, USA; b Pulmonary Fellow, Division of Pulmonary and Critical Care Medicine, BronxCare Health System, Bronx, NY, USA; c Pulmonary Fellow, Division of Pulmonary and Critical Care Medicine, BronxCare Health System, Bronx, NY, NY, USA; d Resident Department of Medicine, BronxCare Health System, Bronx, NY, USA; e Associate Professor, Clinical Medicine, Division of Pulmonary and Critical Care Medicine, BronxCare Health System, Bronx, NY, USA.

**Keywords:** ARDS, coronavirus, COVID-19, COVID-19 waves, mechanical ventilation, mortality, respiratory devices

## Abstract

To expand our limited knowledge of COVID-19-related outcomes in patients admitted to inner-city intensive care unit (ICU across multiple infection waves. This retrospective study compared patients admitted to the ICU in Bronx, NY, during 3 COVID-19 waves (March 2020 to February 2022). Outcomes included in hospital mortality, length of stay (LOS), use of mechanical ventilation, and discharge disposition. The study included 716 patients (343, 276, and 97 in the first, second, and third COVID-19 waves, respectively). The number of days on mechanical ventilation and LOS were lower in the first wave. Of the 345 discharged patients, 37% went home directly, whereas 11% were discharged to a skill nursing facility. More patients went home during the second and third waves. Mortality decreased from the first to the third waves (57%–37%; *P* < .001). Predictors of mortality included age, male gender, COPD, shock, acute kidney injury (AKI), dialysis requirement, and mechanical ventilation. The decreased mortality and better discharge disposition of these inner-city patients during the second and third waves is encouraging, as this population historically had a high COVID-19-related mortality risk.

**Key Points**

• Real-world data on 3 COVID-19 waves with a large cohort of critically ill patients admitted to inner-city Intensive Care Unit.
• The catchment area has the poorest zip code in New York State, with a very high-risk population in the medically underserved area.
• Most of the patients are Hispanics and African Americans with the highest mortality rates reported.

## 1. Introduction

The coronavirus disease 2019 (COVID-19) pandemic is caused by SARS-CoV-2, which has mutated many times since its emergence and has caused multiple “waves” of infection. During the first wave, healthcare system resources were overwhelmed, therapeutic options were limited or nonexistent, the population was largely unprotected, and guidelines cautioned against using steroids and non-invasive ventilatory (NIV) support. During subsequent waves, several factors contributed to the declining incidence of cases, including public health measures such as wearing masks, social distancing, infection control policies, vaccination, and immunity induced by natural infection. Treatment options have changed with the advent of newer therapies, and critical care utilization and practices have continued to adapt based on the guidelines developed between waves. Prior studies have shown that COVID-19 disproportionally affects racial and ethnic minorities and that underserved populations have poorer outcomes.^[1,2]^ The BronxCare Health System serves the South and Central Bronx, which are considered among the poorest in the country. Moreover, these patients represent a diverse population with many comorbidities.

This retrospective study compared the demographic and clinical characteristics and changes in outcome measures in patients admitted to the critical care unit at our institution during one of 3 consecutive COVID-19 waves. In our analysis, we also considered the types of respiratory support device(s) used, patterns of medication, and predictors of mortality.

## 2. Methods

### 2.1. Study design and patients

This retrospective cohort study was conducted at the BronxCare Hospital Center, a 972-bed community teaching hospital serving the South and Central Bronx.

We included patients 18 years or older with a positive nasal swab COVID-19 test (Roche Cobas SARS CoV-2 RNA PCR) admitted to the adult ICU. Patients with a positive COVID-19 test result who were transferred from a noncritical care area to the ICU were also included.

We excluded ICU patients without confirmed COVID-19 and those with confirmed COVID-19 who were admitted to noncritical care units.

Medication management in patients with COVID-19 followed institutional protocols created based on the available literature and national guidelines by the Center for Disease Control and updated as soon as new data were available. The ICU is staffed with a team of eight intensivists who rotate every 2 weeks; treatment protocols and guidelines are followed to provide standardized care as much as possible.

### 2.2. Ethics approval

The study protocol was approved by the Institutional Review Board (approval number 10142104) and followed the amended Declaration of Helsinki. Owing to the retrospective nature of the study, the need for informed consent was waived.

### 2.3. Study period

**Figure 1.**
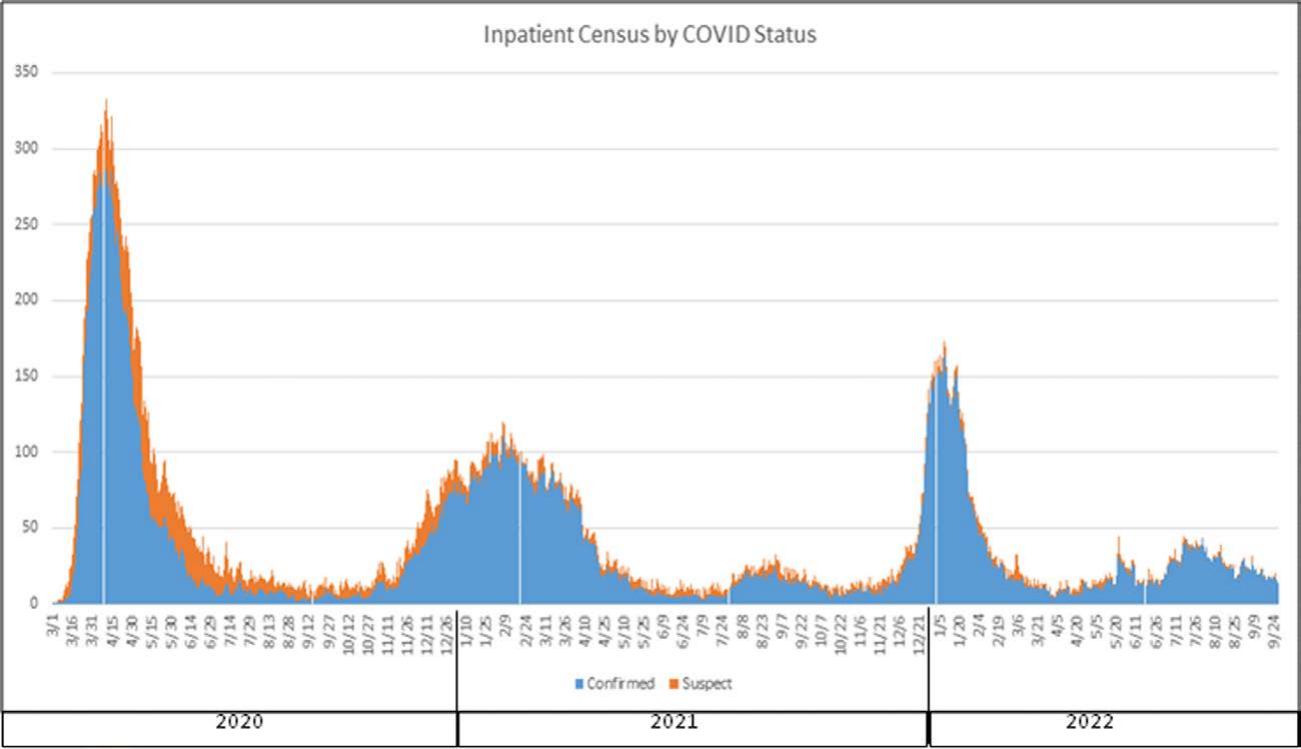
Inpatient census by COVID status. COVID-19 = coronavirus disease 2019.

The first wave at our center occurred from March 13, 2020 to June 2, 2020, the second wave from October 3, 2020to July 1, 2021, and the third wave from December 7, 2021 to February 13, 2022. Figure 1 shows the distribution of patients with COVID-19 admitted to the institution between March 1, 2020, and September 24, 2022.

The primary outcome was all-cause mortality during hospital admission. Secondary outcomes included length of stay (LOS), days of invasive mechanical ventilation (IMV), and discharge disposition. Additional outcomes studied include use of respiratory support devices, and incidence of acute respiratory distress syndrome (ARDS), shock, thromboembolic events, and acute kidney injury (AKI) across the 3 waves. The definition of AKI was based on the KDIGO standards.^[3]^

### 2.4. Data abstraction

All data, including demographic, clinical, and laboratory information, were retrospectively extracted from medical records. Chest radiographs (CXR) were used to collect the radiological findings.

### 2.5. Statistical analysis

We identified 716 patients admitted to the ICU with COVID-19 who met the inclusion criteria. There were 410 (1.193%) missing data points. To establish the nature of the missingness of the data prior to either listwise deletion or imputation, that is, whether the data was Missing Completely at Random (MCAR), Missing at Random (MAR) or Missing Not at Random (MNAR), the Little MCAR test was performed. From the Little’s MCAR test: *χ*^2^(251) = 603.645, *P* = .00. Since the *P* value was <.05, this confirms that the data was not MCAR, but rather MNAR. In this regard, ignoring missing values through listwise deletion or Complete-Case Analysis would result biased estimates in data analysis. Therefore, multiple imputation was considered as the optimal method to handle missing data.

Summary statistics of all variables, including demographic information, preexisting conditions, selected laboratory parameters, and therapeutics used, as well as frequencies and percentages for categorical variables, were obtained. Chi-squared tests were used to compute *P* values to compare the clinical types within the 2 waves, whereas the comparison across all 3 waves was performed using ANOVA. For continuous variables, the means and standard deviations are reported in the tables. The average values of the variables were compared using independent sample *t* tests for 2 clinical types within a group, and the overall *P* value refers to the *F* test using ANOVA, comparing all 3 groups (waves 1, 2, and 3) and the entire cohort.

The major aim of this study was to establish the significant contributing risk factors for mortality in COVID-19 patients across the 3 waves. Because the primary outcome variable (mortality) was measured as a dichotomous categorical variable, the optimal modeling approach was binary logistic regression. Therapeutic agents were excluded from the analysis, as none of the medications were consistently used in all the waves. Patients receiving NIV support by either bi-level positive airway pressure (BiPAP) and/or high-flow oxygen (HFO) were also excluded from the logistic regression analysis, as some patients used one or the other intermittently for comfort and others needed support postliberation from the IMV. The model prediction accuracy was evaluated using Cox and Snell R-Squared and Nagelkerke R-Squared. The *P* values for logistic regression coefficients were computed using Wald’s Z-test coefficients. The significance level was set at α =0.05

## 3. Results

Our study included 716 patients with COVID-19 admitted to the ICU during the 3 waves. All the patients were included in the study; 343 patients in the first wave (March 13, 2020 to June 2, 2020; 82 days), 276 patients in the second wave (October 3, 2020 to July 1, 2021; 271 days), and 97 patients in the third wave (December 7, 2021 to February 13, 2022; 69 days).

Comparisons of patient demographics and preexisting conditions are presented in Table 1. Patients in the last 2 waves were older and predominantly female. More patients with heart failure were admitted to the ICU in the second wave than in the other 2 waves. No differences were observed between the frequencies of the other comorbid conditions across the waves.

**Table 1:** Demographics and comorbid conditions.

Characteristics	Wave 1	Wave 2	Wave 3	Entire cohort	*P* value
	N = 343	N = 276	N = 97	N = 716	
Age (mean ± SD)	60 ± 14	64 ± 14	63 ± 15	62 ± 14	<.001
BMI (mean ± SD)	31.33 ± 9.676	31.01 ± 7.754	27 ± 8	29 ± 8	.001
Gender • Female N (%) • Male N (%)	138(40%) 205 (60%)	162 (59%) 114 (41%)	57 (59%) 40 (41%)	357 (49%) 359 (51%)	<.001
Diabetes	178 (51%)	122 (44%)	45 (46%)	345 (48%)	.15
Hypertension	231 (67%)	198 (71%)	67 (69%)	496 (69%)	.5
CHF	40 (11%)	48 (17%)	23 (23%)	111 (15%)	.008
Asthma	66 (19%)	48 (17%)	11 (11%)	125 (17%)	.19
COPD	43 (12%)	38 (13%)	5 (5%)	86 (12%)	.07
HIV	17 (5%)	16 (5%)	8 (8%)	41 (5%)	.45

### 3.1. COVID-19 complications and laboratory parameters

The analysis of pertinent laboratory parameters (Table 2) showed that patients in the first wave had higher serum lactic dehydrogenase levels, lower serum sodium levels, and higher saturation to fraction of inspired oxygen ratio. Bilateral infiltrates in CXRs were more common in the first 2 waves than in the last wave.

**Table 2:** Lab parameters.

Lab Parameters	Wave 1	Wave 2	Wave 3	Entire cohort	*P* value
	N = 343	N = 276	N = 97	N = 716	
First D-dimer (ng/mL)	3415 ± 8078	3839 ± 9371	3282 ± 9965	3565 ± 8848	.79
Last D-dimer (ng/mL)	6623 ± 18325	4508 ± 16596	2801 ± 7710	5301 ± 16665	.93
First LDH (unit/L)	647 ± 388	595 ± 372	463 ± 372	603 ± 377	<.001
Last LDH (unit/L)	697 ± 1263	657 ± 1667	499 ± 567	656 ± 1379	.49
First sodium (mEq/L)	136 ± 7	136 ± 6	138	137 ± 7	.03
First creatinine (mg/dL)	2.1 ± 3.5	2.3 ± 2.8	2 ± 7.6	2.2 ± 3	.61
Last creatinine (mg/dL)	3.5 ± 3.9	2.5 ± 2.4	2.2 ± 6.4	3 ± 3.3	<.001
First ferritin (ng/mL)	1354 ± 2239	1079 ± 1074	1504 ± 7241	1275 ± 3125	.41
Spo_2_/FiO_2_ ratio on admission	304 ± 152	176 ± 133	281 ± 140	252 ± 155	<.001
CXR • Unilateral • Bilateral Normal	50 (14.58) 270 (78.72) 23 (6.71)	24 (8.7) 238 (86.23) 14 (5.07)	25 (25%) 56 (57%) 16 (16%)	99 (13%) 564 (78%) 53 (7%)	<.001

SpO2/FiO2 = saturation to fraction of inspired oxygen ratio.

The associated COVID-19-related complications and use of ventilatory support can be seen in Table 3. The occurrence of ARDS and AKI was significantly higher in the first and second waves than in the third wave (*P* < .001).

**Table 3:** Critical care diagnosis and ventilator support.

	Wave 1	Wave 2	Wave 3	Entire cohort	*P* value
	N = 343	N = 276	N = 97	N = 716	
Pneumonia	320 (93%)	262 (94%)	81 (83%)	663 (92%)	<.001
Adult respiratory distress syndrome	174 (50%)	172 (62%)	25 (25%)	371 (51%)	<.001
Shock	169 (49.27)	134 (48.55)	38 (39%)	341 (47%)	.19
Thromboembolic disease	20 (5.87)	26 (9.42)	8 (8%)	54 (7%)	.24
Acute kidney injury	229 (66.76)	147 (53.26)	43 (44%)	419 (58%)	<.001
Required hemodialysis	31 (9.04)	46 (16.79)	16 (16%)	93 (13%)	<.05
***Ventilatory support***					
Invasive mechanical ventilator	236 (68%)	154 (55%)	50 (51%)	440 (61%)	<.001
BIPAP	13 (3.8%)	146 (52.9%)	30 (30%)	189 (26%)	<.001
High flow oxygen	27 (7.9%)	156 (56.5%)	39 (40%)	222 (31%)	<.001

BiPAP = bi-level positive airway pressure.

### 3.2. COVID-19 and respiratory support

COVID-19 respiratory infection and use of respiratory support devices are listed in Table 3. Of the 343 patients admitted to ICU in first wave, 320 had pneumonia and 174 developed ARDS. In the second wave 262 of 276 patients were admitted for pneumonia and 172 had ARDS. In the thirds wave 81 of 97 patients were admitted with pneumonia and 25 hadARDS. IMV was used in 68% of the patients in wave 1, 55% of patients in wave 2 and 61% of patients in wave 3. noninvasive ventilation either BiPAP and/or HFO use were higher in wave 2 and low in wave 1.

### 3.3. COVID-19 outcomes

Comparisons of COVID-19-related outcomes among the waves are presented in Table 4. We observed a significant decrease in mortality in the last wave compared with the first 2 waves (57% in the first wave vs 37% in the third wave). The LOS and days on IMV were lower in the first wave.

**Table 4:** Primary and secondary outcomes.

	Wave 1	Wave 2	Wave 3	Entire cohort	*P* value
	N = 343	N = 276	N = 97	N = 716	
Mortality	198 (57%)	132 (47%)	37 (38%)	367 (51%)	.001
ICU length of stay	5.2 ± 5.9	8.3 ± 6.8	5 ± 4.5	6.4 ± 6.2	<.001
Hospital length of stay	12.3 ± 9.7	19.9 ± 17.9	15 ± 11.2	18 ± 11.8	<.001
Days on mechanical ventilation	6.5 ± 5.4	15.1 ± 21.1	11 ± 11.2	10 ± 14.6.	<.001
Discharge disposition Home skilled nursing facility	108 (31%) 37 (10.79)	114 (41%) 30 (10.87)	43 (44%) 13 (13%)	265 (37%) 80 (11%)	.02

Forty-eight percent (345) of patients admitted to the ICU with COVID-19 were discharged from the hospital; 37% went home and 11% were discharged to a skilled nursing facility. More patients were discharged home in the last 2 waves than in the first wave.

### 3.4. Predictors of mortality

The results of our logistic regression analysis for predicting mortality in the entire cohort are presented in Table 5.

**Table 5:** Logistic regression for mortality.

	Odds ratio	CI (95%)	*P* value
Age	1.02	1.02–1.01	<.001
Male gender	2.1	1.6–2.7	<.001
Chronic obstructive airway disease	1.8	1.2–2.6	.003
Admission serum sodium	1.02	1.01–1.04	.001
Last serum creatinine	1.4	1.3–1.5	<.001
Unilateral infiltrates on admission	3.9	2.0–7.6	<.001
Bilateral infiltrates on admission	2.6	1.4–4.8	.001
Shock	6.2	4.6–8.3	<.001
Acute kidney injury	1.7	1.2–2.2	.001
Need for hemodialysis	1.5	1.3–1.8	.01
ICU length of stay	1.1	1.08–1.0	<.001
Hospital length of stay	1.9	1.8–1.9	<.001
Mechanical ventilator	5.1	4.6–5.6	<.001
Days on Invasive mechanical ventilation	1.05	1.03–1.06	.027

ICU = intensive care unit.

Predictors of mortality were older age, male gender, COPD as a comorbidity, sodium levels at admission to the ICU, presence of infiltrates on chest imaging, shock, and AKI with the need for hemodialysis. Other predictors of mortality included the need for IMV, ICU and hospital LOS, and days on MV.

## 4. Discussion

Our comparison of these 3 COVID-19 waves supports the findings of other studies conducted in New York City in that the second wave rose more gradually and had a longer duration than the other 2 waves. Similarly, compared with the prior waves, patients in the third wave showed improvements in several outcomes.^[4,5]^ In contrast to other local studies, we focused only on COVID-19 patients admitted to the ICU rather than other hospital units. Most studies comparing different waves of COVID-19 have shown better outcomes during the later waves, including decreased mortality.^[4,6–9]^ A smaller French study with 132 patients comparing first- and second-wave COVID-19 patients admitted to the ICU in 2021 did not find any significant changes in hospital mortality (50% vs 52%).^[10]^ In our study, despite observing no differences in the frequencies of comorbid conditions and a higher number of patients with ARDS, we noted a steady decrease in hospital mortality in these critically ill patients. The overall hospital mortality rate in our ICU cohort was 51%, with 57% in the first wave and 38% in the third wave. This decrease is likely multifactorial. In the first wave, knowledge of COVID-19 and its clinical course was largely unknown, and the healthcare system was not prepared for the pandemic. In addition, only limited proven therapeutic options were initially available, as steroids, which were later shown to be beneficial, were discouraged.^[11]^ In the initial wave, ICUs and hospitals were overwhelmed by the number of admissions and the need for critical care beds and ventilators compared with the subsequent COVID-19 waves. In later waves, the burden on hospitals and the overall healthcare system decreased as healthcare workers became more familiar with patient management, safety, and infection control protocols, as well as with oxygen support devices in COVID-19 patients. We cannot minimize the effect of COVID-19 vaccination among healthcare providers and the general population, which has led to a decreased number of cases and reduced the burden on critical care resources. Additional explanation for the decreased mortality in subsequent wave is the SARS-CoV-2 mutations and the variants responsible for changing clinical manifestations, change in transmissibility, morbidity and mortality.^[12,13]^ We did see a decrease in incidence of pneumonia, shock requiring vasopressors, need for mechanical ventilation, AKI, thromboembolic disease and ARDS in subsequent waves which may be a major contributing factor in decreasing mortality.

There are many potential explanations for the high mortality rate of critically ill patients with COVID-19. The first is the multi-organ involvement of the disease. Indeed, at least half of our cohort developed shock that required vasopressors, ARDS, or AKI. The development of ARDS in patients with sepsis is independently associated with higher mortality and prolonged hospital and ICU LOS.^[14]^ Hypoxic respiratory failure in patients with COVID-19 has multiple pathophysiological mechanisms and consequences, including a significant risk of thrombotic complications, ranging from microvascular thrombosis and venous thromboembolic disease to stroke. In addition, associated inflammatory cascade dysregulation occurs, which could explain multi-organ failure, including the cardiovascular and renal systems.^[15–17]^

Evidence of medication efficacy (or lack thereof) accumulated during the first wave.^[18–20]^ For example, the use of dexamethasone increased in later waves based on the randomized RECOVERY trial, which showed a lower mortality in patients using dexamethasone and receiving either IMV or oxygen alone.^[21]^ We did not look into therapeutic agents and mortality as changes in therapeutic use across different waves reflected the evolving knowledge and global experiences related to COVID-19, and the use of some drugs, such as dexamethasone, tocilizumab, and remdesivir, increased with time, and some medications like hydroxychloroquine decreased.^[21,22]^

Decreased use of IMV in subsequent waves has been reported in several recent studies.^[4,5,10,23]^ Indeed, we saw a significant decrease in the use of IMV and increased use of NIV compared to the initial wave, supporting the findings of others.^[24]^ In the first wave, BiPAP and HFO therapy were discouraged due to the risk of aerosolization and concerns about the effectiveness of personal protective equipment for healthcare workers, which may have prompted early intubation in more patients on IMV during the first wave. As the pandemic progressed, evolving clinical experience and critical care situational actions resulted in the greater use of BiPAP and HFO. At our institution, patients who declined intubation or failed extubation, leading to adjusted oxygenation protocols, were administered BiPAP or HFO with additional support of HEPA filters and placed in a respiratory isolation room with exchangers.

Contrary to Belgian and French studies, our patients stayed longer on IMV and had a greater ICU and hospital LOS in the last 2 waves than in the first.^[9,10]^ This finding could be explained in that we had more patients with ARDS in our second wave who required IMV, and ICU beds were not scarce at our hospital.

Predictors of mortality have been evaluated in many studies. An earlier meta-analysis revealed poor outcomes in older patients, higher APACHE II and SOFA scores, and a lower PaO_2_/FiO_2_ ratio, whereas clinical interventions such as IMV, kidney replacement therapy, and vasopressors have been used to improve survival.^[25,26]^ Other reported predictors of mortality are the need for IMV, days on mechanical ventilation, and hospital LOS.^[27,28]^ Multiple studies have shown that mortality in ARDS patients is approximately 40%.^[29–32]^ Similarly, in our study, older age, shock, the need for IMV, AKI, the need for hemodialysis, and COPD as a comorbid condition predicted mortality.

Our study has several strengths. This is one of the largest descriptive studies from a single center evaluating patients admitted to the ICU during 3 consecutive COVID-19 waves. This study included patients from an inner-city population, representing minority groups that are usually underrepresented in many studies. Our hospital serves mainly African Americans and Hispanics, who have had the highest mortality among all racial and ethnic groups due to COVID-19.^[33,34]^ Their reported mortality rate due to COVID-19 varies from 26% to 78%.^[35–37]^

The main limitation of the study is that, despite its large cohort of 716 critically ill patients, this was a retrospective study performed in a single center, with the resultant bias associated with any similar type of study. Due to the retrospective nature and data abstraction from the electronic medical record, we could not separate patients based on the different modalities used for NIV; some patients were supported by alternating modalities for comfort, or NIV was used postliberation for IMV. We could not extract the vaccination history in the subsequent waves.

We only included patients admitted to the ICU to minimize variation in other aspects of care. During the first wave, many patients received critical care outside the ICU from non-intensive-care-trained physicians or nurses with a wide range of monitoring capabilities and a frequent lack of resources to evaluate adherence to established protocols.

Although variants of the original SARS-CoV-2 virus have emerged throughout the pandemic, data indicate that the initial wave was due to an ancestral variant, followed by the delta variant in the second wave, and the omicron variant in the third wave. We did not routinely perform SARS-CoV-2 genotyping among patients, nor did we reliably determine the patients’ vaccination status.

## 5. Conclusions

The COVID-19 pandemic has exposed the vulnerability of our existing medical system, but at the same time, it has demonstrated our capability to evolve, learn, and implement changes rapidly to more successfully treat critically ill patients. The second and third COVID-19 waves in our Bronx population exhibited a steady decrease in mortality rates, with more patients discharged home instead of skilled nursing facilities than patients in the first wave. this improvement in outcomes may reflect medical advances, such as earlier diagnosis, better use of new therapies, improved public health measures, such as vaccination, and the skills of critical care team to learn, unlearn and relearn as new data and findings constantly published, to provide care to these groups of patients. In addition, the changing virulence of SARS-CoV-2 mutants with a resultant changing clinical manifestations played a major role.

## Acknowledgments

The authors would like to thank the BronxCare Health System Information Technology Department for their contribution in generating institutional data since the beginning of the pandemic. We also thank Dr Christian Mendoza for assisting with partial acquisition of data.

## Author contributions

**Conceptualization:** Sindhaghatta Venkatram, Gilda Diaz-Fuentes.

**Data curation:** Sindhaghatta Venkatram, Arundhati Dileep, Ked Fortuzi, Nishant Allena.

**Formal analysis:** Sindhaghatta Venkatram, Arundhati Dileep, Ked Fortuzi, Nishant Allena, Gilda Diaz-Fuentes.

**Investigation:** Gilda Diaz-Fuentes.

**Methodology:** Sindhaghatta Venkatram, Gilda Diaz-Fuentes.

**Software:** Sindhaghatta Venkatram, Nishant Allena.

**Writing – original draft:** Sindhaghatta Venkatram, Gilda Diaz-Fuentes.

**Writing – review & editing:** Sindhaghatta Venkatram, Arundhati Dileep, Ked Fortuzi, Nishant Allena, Gilda Diaz-Fuentes.
